# Temporal Pattern of micro-CT Angiography Vascular Parameters and VEGF mRNA Expression in Fracture Healing: a Radiograph and Molecular Comparison

**DOI:** 10.1055/s-0042-1757466

**Published:** 2023-01-30

**Authors:** Aga Satria Nurrachman, Azhari Azhari, Lusi Epsilawati, Farina Pramanik

**Affiliations:** 1 Department of Oral and Maxillofacial Radiology, Faculty of Dental Medicine, Universitas Airlangga, Surabaya, Indonesia; 2 Department of Dentomaxillofacial Radiology, Faculty of Dentistry, Padjadjaran University, Bandung, West Java, Indonesia

**Keywords:** fracture healing, micro-CT angiography, VEGF mRNA expression, temporal pattern

## Abstract

Angiogenesis plays an important role in fracture healing with vascular endothelial growth factor (VEGF) as the main protein involved. Micro-computed tomography (CT) angiography may be used to analyze this revascularization with several parameters such as number of branches, total volume, and diameter. This systematic review is aimed to assess available studies on the temporal pattern of vascular imaging on micro-CT angiographs, especially in terms of the number of branches, total volume, and diameter as well as the temporal pattern of VEGF mRNA expression as the molecular comparison during bone fracture healing. This review was conducted according to the PRISMA guidelines. Electronic database searches were performed using PubMed, ProQuest, ScienceDirect, EBSCOhost, Taylor & Francis Online, and hand searching. The search strategy and keywords were adjusted to each database using the Boolean operators and other available limit functions to identify most relevant articles based on our inclusion and exclusion criteria. Screening and filtration were done in several stages by removing the duplicates and analyzing each title, abstract, and full-text in all included entries. Data extraction was done for syntheses to summarize the temporal pattern of each parameter. A total of 28 articles were eligible and met all criteria, 11 articles were synthesized in its angiograph's analysis, 16 articles were synthesized in its VEGF mRNA expression analysis, and 1 article had both parameters analyzed. The overall temporal pattern of both three micro-CT angiographic parameters and VEGF mRNA expression was in line qualitatively. The number of branches, total volume, and diameter of the blood vessels in micro-CT angiography showed an exponential rise at week 2 and decline at week 3 of fracture healing, with the VEGF mRNA expression concurrently showing a consistent pattern in the phase.

## Introduction

Fracture is a bone discontinuity caused by extreme physical pressure or trauma, which can cause bone displacement and is typically accompanied by injury to blood vessels and surrounding soft tissue. According to the 2013 Global Burden of Injury report, the incidence of fracture cases is identified in ∼21.7 million people receiving inpatient services and ∼108 million people receiving outpatient services. 1 In Indonesia, the overall incidence of injury cases has climbed from year to year, with 7.5% cases in 2007, 8.2% in 2013, and 9.2% in 2018. The study also discovered that the lower extremities, upper extremities, and craniofacial area are the most affected region by injuries. 2 Fractures of the mandible, maxilla, nasal, and zygoma were the most common fracture cases recorded in the craniofacial area, with mandibular fractures accounting for 36 to 59% of all cases. 3 4 5 6 7 8

Bone is an organ consisting of many blood vessels. In fractures, damage also occurs to the blood vessels. Angiogenesis or the formation of new blood vessels has an important role in the bone healing process, both in endochondral and intramembranous ossification. Angiogenesis occupies a large part of the bone regeneration process after fracture compared with other processes. The angiogenesis process can pave the way for progenitor cells that form bone, nutrients, oxygen, and other minerals to arrive at the fracture site and start the remodeling process. 9 10 11 12 Vascular endothelial growth factor (VEGF) is the main mediator protein in the process that acts specifically on endothelial cells. Hypoxic conditions in the fractured tissue are known to activate the HIF-1α signaling pathway, which will trigger an increase in the VEGF mRNA at the cellular level before VEGF is then secreted into the tissue. 13

Radiological examination is the most commonly objective procedure used by clinicians in detecting and evaluating the fracture healing process. Micro-computed tomography (micro-CT ) is a high-resolution radiographic modality, equivalent to CT, which was developed specifically for microstructural studies of small objects. The application of micro-CT has been developed by many researchers to be used as a tool for analyzing soft tissue, including vascular structures in experimental animals. 14 15 16 17 18 The use of certain contrast agents, such as barium sulfate or Microfil, is required so that blood vessels can be visualized properly, namely micro-CT angiography. 19 20 21 22 23

Studies on the correlation between VEGF and vascular angiographic parameters in fracture healing are still minimal and difficult to find in various databases. Most of the existing studies only assessed vascular morphology and VEGF separately or in non-specific applications such as without using radiographs and did not focus on the healing process of bone fractures. The description of the overall pattern of angiogenesis in the fracture healing process, including the direct correlation between these two parameters, is something that has not been studied in depth. This review is aimed to pre-assess this correlation by comparing the two parameters to be a more valid reference of angiogenesis characteristics that occurs during the physiological healing of bone fractures as a basis for further development and research in fracture management, particularly for craniofacial fractures.

## Methods

### Research Question

The research question was modified using PICO model, its P (Problem) is the fracture healing process, I (Intervention) is the micro-CT angiography and RT-PCR VEGF, and O (Outcome) is the temporal pattern of three micro-CT angiography parameters and the RT-PCR VEGF mRNA expression, while there is no exact C (Comparison) in this review. The detailed outcome for micro-CT angiography in this study was limited to three parameters: (i) the number of branches; (ii) total volume; and (iii) diameter of the blood vessels, as a result of the analysis.

### Literature Search

Search strategy was performed using five electronic databases: PubMed, ScienceDirect, ProQuest, EBSCOhost, and Taylor and Francis Online using the keyword *((((((((fracture) AND (healing)) AND (bone)) AND (vascular)) AND (micro-ct)) OR (vegf mrna expression)) AND (rt-pcr)) NOT (tumor)) NOT (cancer)* following the concept of Boolean operators and the available limit functions in each database to identify relevant articles. Hand searching was done additionally to get any supplemental findings meeting the eligibility criteria. The search of all published studies was performed in September 2020 and updated in November 2020.

### Inclusion Criteria

Studies must meet these following inclusion criteria: (i) type of study: all experimental and observational RCTs; (ii) type of sample: mice or rats; (iii) focused on fractur healing; (iv) available in full-text version; (v) available in English; and (vi) published in 2010–2020.

### Exclusion Criteria

The identified entries were excluded based on these following criteria: (i) studies did not have a fracture group in normal conditions without any additional interventions or treatments except fixation, or modified pathological and systemic pre-conditioning; (ii) results did not provide any temporal pattern of micro-CT imaging or VEGF mRNA expression within certain time point of healing process; (iii) for VEGF studies, total RNA was not extracted directly from fracture tissues, the non-intervention group became the comparison group when calculating the multiplication in the RT-PCR analysis, and the assessment of mRNA expression was performed on VEGF isoforms or other VEGF derivate groups.

### Data Extraction and Synthesis

The articles identified through the search strategy were filtered gradually following the PRISMA (Preferred Reporting Items for Systematic Reviews and Meta-analyzes) concept by examining the relevance of the title, abstract to full-text based on the inclusion and exclusion criteria. Data analysis and synthesis were performed qualitatively by integrating all the included studies data using thematic analysis that aimed to produce a conceptual theory (meta-ethnography) about the temporal patterns of all predetermined fracture healing parameters.

### Risk of Bias Assessment

The final entries were assessed using the SYRCLE risk of bias tool as this review is focused on animal studies. Each aspect of the assessment was formed as 10 signaling questions according to the guidelines to be answered by the reviewers with “Yes,” “No,” and “Unclear” answers. “Yes” answer indicated a low risk of bias, “No” answer indicated a high risk of bias, and an “Unclear” answer indicated the risk of bias that was unclear or there was insufficient or detailed information to determine the risk of bias correctly. Questions 1 to 3 represented the aspect of selection bias, questions 4 and 5 represented performance bias, questions 6 and 7 represented detection bias, question 8 represented incomplete outcome data, question 9 reported bias and question 10 represented other biased judgments. The disagreements between researchers for each assessment were resolved by joint discussion to reach a consensus or by consulting with a third person. 24

## Results

**Fig. 1:**
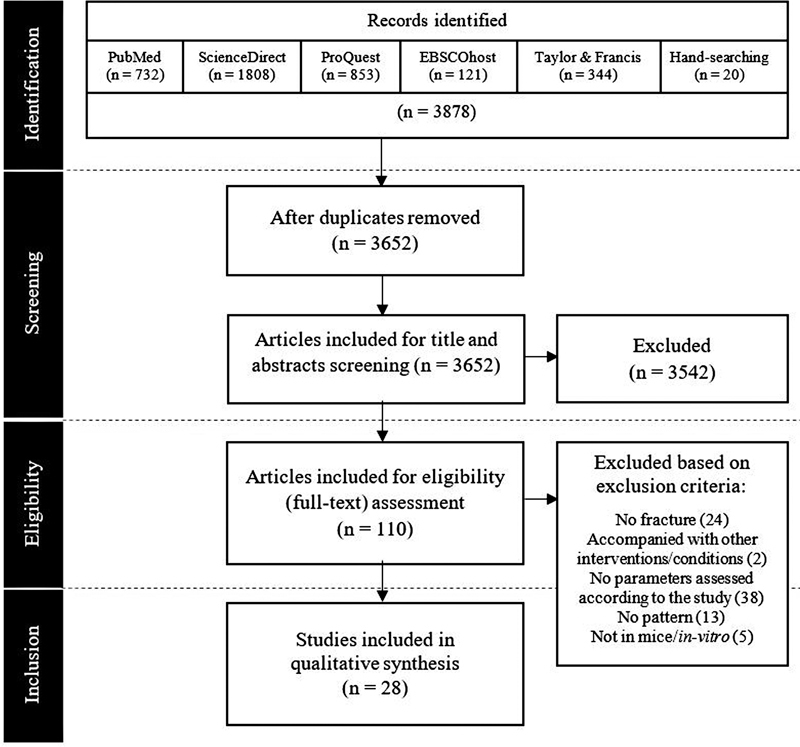
PRISMA flow diagram for article identification and screening.

A total of 3,878 articles were identified and underwent various stages of screening with 226 articles as duplicates; 3,542 articles were removed during the screening phase and 82 articles were removed during eligibility assessment. A final of 28 articles were included for further processing, of which 16 articles assessed micro-CT angiographic patterns, 11 articles analyzed VEGF mRNA expression patterns, and 1 article assessed both. The screening flow is outlined in the PRISMA chart in Fig. 1 . All included articles had the earliest publication year as 2010 and the latest as 2019. Of all 17 micro-CT studies, 5 articles assessed the number of vascular branching, 16 articles assessed the total volume, and 5 articles assessed the vascular diameter. A list of all included studies is presented in Table 1 .

**Table 1:** List of included studies

Author (publication year)	Title	Aim
Chen et al 25 (2017)	*GIT1* gene deletion delays chondrocyte differentiation and healing of tibial plateau fracture through suppressing proliferation and apoptosis of chondrocytes	Compare the healing process of tibial fractures in *GIT1* KO and normal conditions
Cheung et al 26 (2012)	Stimulated angiogenesis for fracture healing augmented by low-magnitude, high-frequency vibration in a rat model—evaluation of pulsed-wave Doppler, 3D power Doppler ultrasonography, and micro-CT microangiography	Determine the effect of vibration therapy on angiogenesis in fracture healing, especially in osteoporosis conditions
Cottrell et al 27 (2014)	BMP-2 modulates the expression of other growth factors in a rat fracture healing model	Investigate the role of local BMP-2 therapy in fracture healing
Ding et al 28 (2010)	Changes in substance P during fracture healing in ovariectomized mice	Assess the role of substance P during fracture healing in osteoporosis
Ding et al 29 (2011)	Bone loss and impaired fracture healing in spinal cord-injured mice	Compare the fracture healing process in spinal cord injury
Gilbert et al 30 (2015)	Contaminated open fracture and crush injury: a murine model	Assess the fracture healing process in bacterial-infected conditions
He et al 31 (2012)	Deletion of estrogen receptor β accelerates early stage of bone healing in a mouse osteotomy model	Compare the healing process of the estrogen receptor-KO fracture
He et al 32 (2011)	Impaired bone healing pattern in mice with ovariectomy-induced osteoporosis: a drill-hole defect model	Apply the drill-hole bur defect technique as a model of bone healing
Hurley et al 33 (2016)	Accelerated fracture healing in transgenic mice overexpressing an anabolic isoform of fibroblast growth factor 2	Investigate the expression of genes involved in the fracture healing process of FGF2 transgenic mice
Kidd et al 34 (2010)	Temporal pattern of gene expression and histology of stress fracture healing	Investigate the temporal pattern of gene expression and the histology of fracture healing processes due to repetitive stress
Li et al. 35 (2019)	GIT1 regulates angiogenic factor secretion in bone marrow mesenchymal stem cells via the NF-κB/Notch signaling to promote angiogenesis	Compare fracture healing angiogenesis in *GIT1* KO and normal (wild-type) conditions
Li et al 36 (2012)	Expression of *VEGF* gene isoforms in a rat segmental bone defect model treated with EPCs	Evaluate *VEGF* gene expression after local EPC therapy
Li et al 37 (2018)	Neurotrophin-3 improves fracture healing in rats	Investigate the role of neurotrophin-3 in fracture healing
Liu et al 38 (2017)	Exogenous hedgehog antagonist delays but does not prevent fracture healing in young mice	Evaluate the effect of GDC on fracture healing
Macdonald and Shefelbine 15 (2013)	Characterizing neovascularization in fracture healing with laser Doppler and micro-CT scanning	Compare the doppler laser and micro-CT in assessing post-fracture vascularization
Martinez et al 39 (2010)	Healing of non-displaced fractures produced by fatigue loading of the mouse ulna	Observe the fracture healing process created with the new protocol (fatigue loading)
Matsumoto et al 40 (2013)	Subtraction micro-computed tomography of angiogenesis and osteogenesis during bone repair using synchrotron radiation with a novel contrast agent	Test the use of new contrast agent zirconium dioxide (ZrCA) in the assessment of angiogenesis and bone healing
McCabe et al 41 (2013)	Simulated microgravity alters the expression of key genes involved in fracture healing	Investigate the effect of low gravity on gene expression involved in fracture healing
Minkwitz et al 42 (2015)	Longitudinal analysis of osteogenic and angiogenic signaling factors in healing models mimicking atrophic and hypertrophic non-unions in rats	Analyze fracture healing in atrophy and hypertrophic healing model conditions
Qiao et al 43 (2019)	Comparison of remote ischemic preconditioning and intermittent hypoxia training in fracture healing	Compare fracture healing in preconditioning ischemia and hypoxia
Reumann et al 44 (2010)	Production of VEGF receptor 1 and 2 mRNAs and proteins during endochondral bone repair is differential and healing phase-specific	Examine the expression of VEGF and protein during the fracture healing process in the ribs
Suen et al 45 (2014)	Sclerostin monoclonal antibody enhanced bone fracture healing in an open osteotomy model in rats	Evaluate the administration of sclerostin on the bone healing process
Sun et al 46 (2012)	Three-dimensional, high-frequency power Doppler ultrasonography for the assessment of microvasculature during fracture healing in a rat model	Compare the use of high-frequency power doppler 3D ultrasound with micro-CT angiography for microvascular analysis
Wang et al 47 (2012)	Accelerated calvarial healing in mice lacking Toll-like receptor 4	Investigate the bone healing process in TLR4-KO mice with wild-type mice
Wilson et al 48 (2015)	Expression of angiopoietin-like protein 4 at the fracture site: regulation by hypoxia and osteoblastic differentiation	Examine Angptl4 expression at the fracture site
Yin et al 49 (2014)	Impaired angiogenesis during fracture healing in GPCR Kinase 2 interacting protein-1 (GIT1) knock out mice	Compare the fracture healing process in wild-type (normal) with GIT1 KO mice
Yuasa et al 50 (2014)	The temporal and spatial development of vascularity in a healing displaced fracture	Propose a new theoretical model in the process of revascularization of post bone fractures
Zhao et al 23 (2014)	Effect of fixation on neovascularization during bone healing	Investigate the effect of fixation using a new external fixator on the angiogenesis of the fracture healing process

**Fig. 2:**
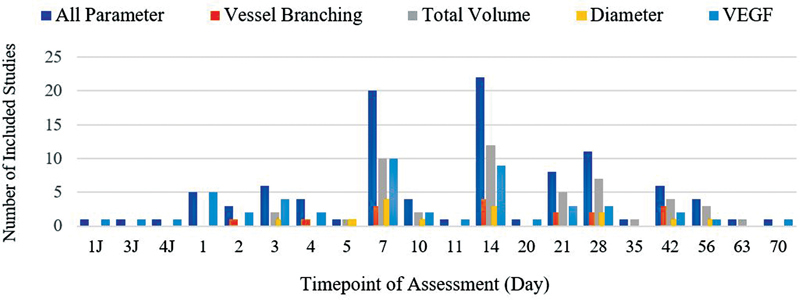
Timepoint of assessment in included studies.

The included studies had variations in the selection of the assessment time points. Most of the included articles had assessed at days 7, 14 and 28 post fracture as depicted on the graph in Fig. 2 . The samples used in all included studies were mice and rats with different species, with the majority using C57BL/6 inbred laboratory mice and Sprague–Dawley rats ( Supplementary Table S1 , available in the online version only). Based on the fracture type, most studies used the open or closed transversal fracture model. There were several fracture conditioning methods performed on experimental animals ranging from using a fracture apparatus, burs, saws, scissors, with some remaining undescribed. Long bones became the common fracture site, with the most frequent areas selected were the femur and tibia. Most articles described the use of fixation post fracture, ranging from Kirschner wire, needle, pin or external fixation, with others having no clear description ( Supplementary Table S2 , available in the online version only).

Of a total of 17 inclusion studies that performed micro-CT angiographic assessments, 14 used the Microfil contrast agent to visualize vasculature ( Supplementary Table S3 , available in the online version only). Nearly all samples were decalcified prior to micro-CT imaging. The micro-CT resolution used in the studies varied, ranging from as little as 2.74 to as large as 21 µm. There were several selectable imaging areas (scans) and volume of interest (VOI) but most studies did not clearly describe them. Some studies also did not explain the method of micro-CT angiograph data processing, especially regarding the reconstruction and analysis software used, including the detailed methods regarding segmentation and analysis performed in calculating the vascular morphometry. Most of the included studies used the reverse transcriptase-polymerase chain reaction (RT-PCR) analysis with the relative expression ΔΔCτ method that analyzed the results by normalizing the data with internal controls and then comparing them with the non-fracture group or the normal group ( Supplementary Table S4 , available in the online version only).

**Fig. 3:**
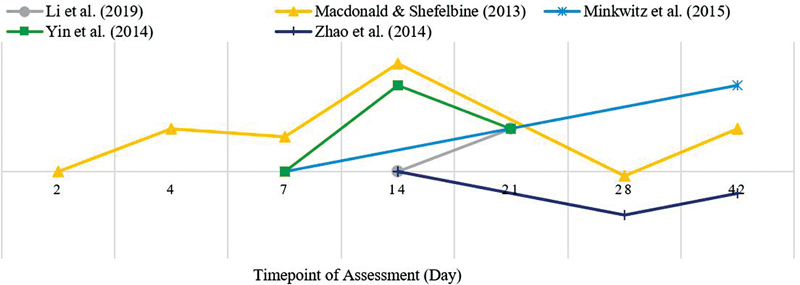
Temporal pattern of vessel branches in micro-CT angiograph.

**Fig. 4:**
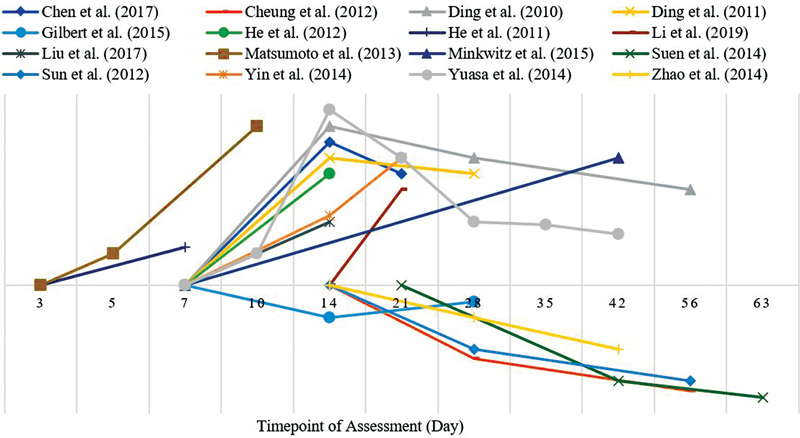
Temporal pattern of total volume in micro-CT angiograph.

**Fig. 5:**
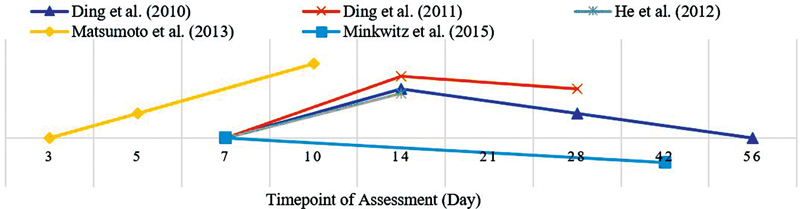
Temporal pattern of vessel diameter in micro-CT angiograph.

**Fig. 6:**
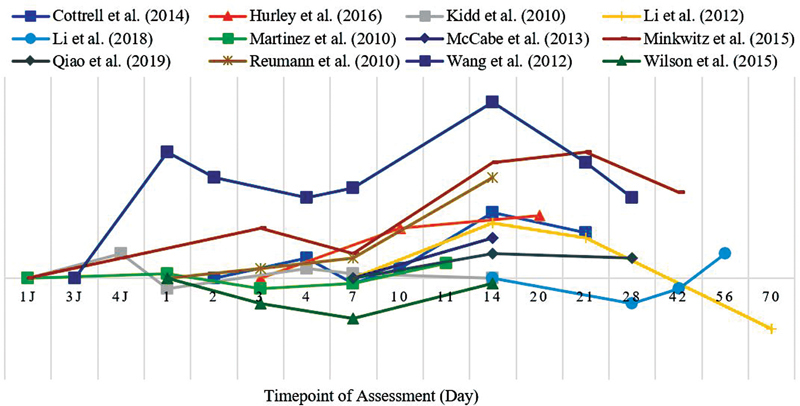
Temporal pattern of VEGF mRNA expression.

The results of the analysis of micro-CT angiographic images of each parameter and the VEGF mRNA expression in this review were synthesized qualitatively and presented in the form of pattern curves based on changes that occurred according to the fracture healing time points listed in all included articles. The curve presented only represented a picture of the increase or decrease in qualitative terms from the results of the assessment performed at the relevant point in time, without any specific numerical standardization or the relationship between the assessment result data between articles. The curves of the temporal pattern that occurred during the fracture healing process on the number of blood vessel branching are presented in Fig. 3 , total vascular volume in Fig. 4 , and diameter in Fig. 5 , while the temporal pattern of changes in the VEGF mRNA expression is presented in Fig. 6 .

**Fig. 7:**
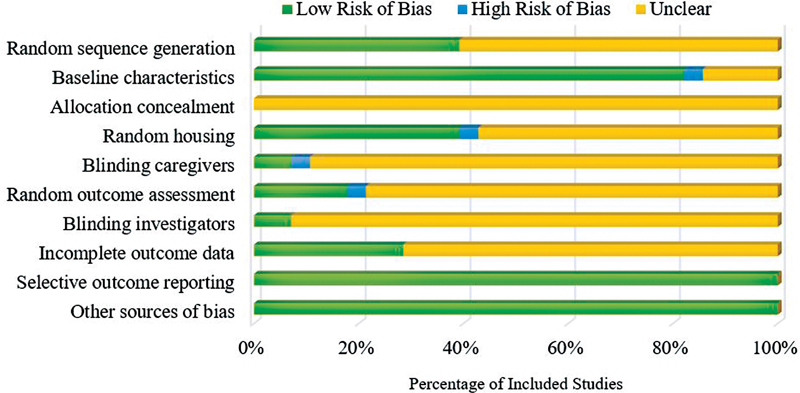
Result of the SYRCLE risk of bias assessment.

The results of the risk assessment of bias generally have an unclear risk of bias ( Fig. 7 ) with as many as 85% of studies (24 articles) having a “Yes” rating on ≤ 5 aspects of assessment (low risk of bias) and 78% of the studies (22 articles) had an “unclear” score on ≥ 5 aspects of the assessment (risk of unclear bias) out 28 articles. The lowest bias rating in this review was found in ∼14% of the studies (4 articles) with the ratio of “Yes” and “Unclear” being 6:4.

## Discussion

Laboratory mice C57BL/6 and Sprague–Dawley rats, with an age range of 12 to 24 weeks were widely used as research subjects by most of the included studies that were reportedly as models of adult humans aged 20 to 30 years. 51 52 Studies with mice and rats have been found to be credible for various studies at the molecular level, including in studying the regeneration processes of bone *in vivo* because it has physiological characteristics similar to that of human bones. 53 54 55 Long bones were often chosen as a model in various fracture studies using rat experimental animals because they are considered to have a cross-sectional size large enough to be observed, especially using micro-CT, are easy to perform stable fixation, and have been representative used in various studies as a model of bone fracture in general. 55 56 57 Most micro-CT studies performed analyses using the total volume parameter (16 articles) rather than the number of branches (5 articles) or the diameter (5 articles). This is probably because volume measurement is the baseline assessment and is the easiest analysis on micro-CT compared with the other two parameters, which still have to go through other additional pre-processing methods after segmentation. 17

**Fig. 8:**
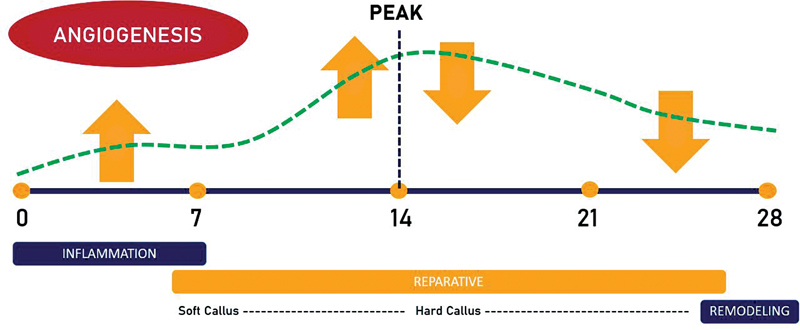
Proposed temporal pattern of angiography micro-CT and VEGF in fracture healing.

In this review, the authors did not identify any studies that correlated directly the relationship between micro-CT angiographic analysis parameters and VEGF mRNA expression values during fracture healing. Although it could not be correlated significantly and quantitatively, it appears that the VEGF value with the three parameters of micro-CT angiography shows a consistent pattern during fracture healing, an increase pattern around week 2, which is then followed by a gradual decrease days after ( Fig. 8 ). The results obtained were as expected and indeed consistent with various theories in previous scientific publications where in the fracture healing process, angiogenesis was found to be most active and significant around the inflammatory and reparative phases, especially around days 7 to 14 and then it will begin. It decreased after 14 days when the healing process had entered the hard callus and remodeling phase. 15 30 58 59 60 61

Nakatsu et al reported that there was an association of VEGF with vascular morphometry, in which several VEGF isoforms such as VEGF121 and VEGF165 induce an increase in the diameter, number of branches, and total vascular length during angiogenesis in *in vitro* models. Apart from its role by increasing the vascular permeability, VEGF was also found to tend to increase the diameter of blood vessels through proliferation and increase in the number of endothelial cells (hyperplasia) in newly formed vascularization and not the result of enlargement of endothelial cells (hypertrophy). 62 This is also in line with Stiver et al who reported that VEGF triggers the enlargement of the vascular lumen accompanied by the appearance of endothelial cells that are actively mitigating and proliferating around them through immunohistochemical examinations. 63 There have been many studies that prove that VEGF plays a role in stimulating the proliferation, differentiation, migration, and activation of endothelial cells as well as increasing the vascular permeability. This picture of the enlargement of the diameter of the blood vessels is also accompanied by the formation of branches and an increase in the total vascular volume, which is the result of the ongoing process of angiogenesis. 9 11 12 64 65 66

VEGF plays a major role in stimulating angiogenesis and is very important in the bone healing process. VEGF was found to work in almost all stages of bone healing from the inflammatory, reparative, ossification, to the remodeling phase. Research on human subjects showed that VEGF levels in post fracture hematomas increased sharply, which was 15 times greater than VEGF levels in the plasma of the control group. This study suggests that the hematoma, which is a fibrin matrix deposition that occurred within hours to days post fracture, acts as a VEGF reservoir. 67 68 In this inflammatory phase, VEGF is concentrated in the hematoma in response to hypoxic conditions that occur. VEGF at this stage plays a role in the formation of granulation tissue, works by triggering chemotaxis of neutrophils and other inflammatory cells and increasing vascular permeability. In the resolution phase or around 3 to 7 days post fracture, these neutrophils trigger macrophages to change the direction of the healing process, secrete several mediators such as TGF-β1, and suppress the inflammatory response to initiate the reparative phase. 65 VEGF is predominantly secreted by hypertrophied chondrocytes, and the secretion is increasingly triggered by the callus avascular condition through the HIF-1α signaling pathway when it enters the reparative phase, especially in the soft callus stage. The VEGF is then tasked with stimulating vascular invasion and calling osteoclasts into the callus. 65 This review shows a significant increase in all micro-CT angiographic parameters and VEGF mRNA expression that occurs on days 7 to 14 post fracture, which is probably closely related to the phase where angiogenesis is the most active.

Between reparative and remodeling phases, VEGF then induces angiogenesis to form the bone through its communication with bone morphogenetic protein (BMP) and direct activation of osteoblast cells. 12 69 Vascular formation through the angiogenesis process at this fracture site forms a pathway for bone progenitor cells to arrive and trigger the calcification of the extracellular matrix in the callus. Entering the hard callus phase, VEGF expression slowly decreases after reaching its significant concentration, as shown in the included studies in this review after the day 14 post fracture, which is probably due to the formation of vascularization in the bone so that oxygen and other nutrients may adequate. VEGF plays an autocrine and paracrine role in invading the vascular into the callus, which stops and changes the program originally intended for callus formation to become a bone formation program. 67 This is confirmed by the study of Yuasa et al where the process of revascularization at the fracture site was found to be inversely proportional to the size of the soft callus and the size of the hard callus. 50

There is data heterogeneity of the included studies which is not measured quantitatively in this study, such as various animals used, fracture methods, micro-CT angiography methods, and methods of RT-PCR analysis. The experimental animal samples varied considerably based on age, sex, and number of samples. Age is one of the factors that need to be considered in animal samples where it can represent a certain age in humans. 52 Various literature has conducted studies on the age of mice and compared it with adult human ages. 70 The oldest age included in this review is the study by Qiao et al, 43 which used 72 to 80 weeks aged rats for human model of the elderly. In older rats, it was also reported that there was a decrease in their locomotor activity and limited tissue repair and regenerative capacity, which may affect the fracture healing process. 71 72 The sex also varied with 11 studies using male rats, 12 female rats, 1 mixture, and the remaining 4 had no description. Differences in the morphology and microenvironment in the body and bone tissue between the sexes may influence the fracture healing process. 73 The number of samples used in the included study varied considerably from 2 to 18 rats per time point of the assessment. Most of the included studies also did not explain the sample size calculation method used in detail. The number of samples itself is vital in the design of a study where too few or too many samples are used that can affect the results or outcome of the study. 74

Based on the method used in micro-CT angiography examination, there is heterogeneity in determining the resolution or size of isotropic voxels. The resolution used in the included studies varied from 2.74 µm to 21 µm. Almost all studies did not have a major focus on the role of micro-CT so there were no detailed descriptions of the volume of interest (VOI), reconstruction methods, and micro-CT angiograph image analysis, including in-depth explanations related to the stages or segmentation processes as well as determining the threshold to separate the image of blood vessels from bone tissue for analysis. As many as 12 of the 17 studies were included although they mentioned the threshold treatment and segmentation method used, there were still no clear details. In terms of contrast agent used, there were two studies using different contrast agents. All these aspects were closely related to the final results. 17 75 76 Meanwhile, there were variations in the RT-PCR reading method of the VEGF mRNA expression where the relative expression ΔCτ method was more widely applied. In addition, the types of internal controls used also varies, such as GAPDH, β-actin, r18s, Hprt, and Ppia, each of which has different characteristics so that it might affect the final threshold value. 77

This review synthesized data qualitatively on specified outcomes. There were no numeric data recording and quantitative processing including the calculation of the statistical significance of changes obtained between time points in each study, the correlation between the included studies, and between parameters and the degree of study heterogeneity. This review also focused on studies using experimental animals due to the large number of available publications compared with human studies in its application for the observation of an *in vivo* bone fracture healing process. The role of a systematic review based on experimental animal studies has limitations, that the results are considered not reliable enough to represent the outcomes that occur in humans so that generally it tends to be used only as a basis for the development for further preclinical and clinical research rather than being able to directly contribute to human health. 78

Caution must also be exercised in interpreting these data, as none of the research included in this study employed the craniofacial bone as a fracture conditioning site, while the majority used long bones such the femur and tibia. During the full-text eligibility stage of review, there were two articles that focused on the vascular analysis of mandibular fractures but did not present results in the form of temporal patterns of changes in micro-CT angiographic images over time and were thus excluded from our review. 79 80 Thus, these findings might not be completely representative of the condition of fracture healing in the craniofacial region as fracture healing is a complex series of regenerative mechanisms that occur simultaneously with two different osteogenesis mechanisms, endochondral ossification, and intramembranous ossification. Endochondral ossification is known as the most common in long bones such as the femur and tibia in which mesenchymal cells differentiate and proliferate into chondrocytes, fibroblasts, osteoprogenitors, and osteoblasts, which then secrete angiogenic factors and trigger ossification toward the epiphysis by forming soft callus as a guide for the bone regeneration process, whereas intramembranous ossification generally occurs and is more dominant in flat bones such as the craniofacial bone in which mesenchymal cells develop straight to produce rows of osteoblasts that congregate locally to form ossification centers and secrete the extracellular matrix that stimulates the production of new bone. 11 12

However, some studies stated that most fractures heal with a combination of both intramembranous and endochondral ossification. 81 82 Another study also discovered that the bone repair of facial bone fractures is mostly produced by endochondral processes, implying that direct bone repair is less common as it requires a perfect reduction and compression of the fracture with a distance between the bone segments of less than 0.1 mm. 83 Furthermore, the movement at the fracture site must be limited as direct consolidation of the bone necessitates rigorous immobilization for the fragile medullary vessels to penetrate through the necrotic bone and the fracture site. Based on this evidence, the distance of fracture fragments and post-fracture stabilization or immobilization might have a greater influence on the occurrence of differences in the fracture healing process than the type or shape of the bone, thus we continued to conduct the review despite the inevitable circumstance of the use of long bones as the fracture sample in all included studies as it is still in the scope and closely related to craniofacial fractures. Nonetheless, this work has highlighted that the blood vessel morphology analysis in angiography using the advanced radiographic modality of micro-CT and the VEGF mRNA expression using RT-PCR were both shown in line during the healing phase.

The limited information provided by researchers in the included studies was a major problem so that most studies had unclear risk of bias. This is consistent with reports in several other systematic reviews that also applied the SYRCLE method for assessment. Published experimental animal studies to date still have poor and detailed writing quality, especially in terms of daily treatment or care (housing conditions) to the data collection process (outcome assessment). 24 Included studies did not provide descriptions in terms of randomization and blinding techniques performed during the study, both when grouping samples into groups, during treatment or daily treatment including lighting and cage temperature, up to the time of assessing the results assessment so that the risk of bias could not be determined with certainty using existing tools. Some included studies only mentioned randomization at several stages of research where the assessors might categorize them as having a low risk of bias, but all these studies still did not provide detailed descriptions or any actual evidence of the randomization process that had been performed during research. Improvement of the quality of writing and reporting of research articles with studies on experimental animals is needed.

## Conclusion

Based on the review of all included studies that have been performed, the following conclusions are obtained: (i) The temporal pattern of blood vessels in micro-CT angiography, including the number of branches, total volume, and diameter of the blood vessels increases at week 2 (days 7–14) and decreases at week 3 (days 15–28) in fracture healing; and (ii) the temporal pattern of VEGF mRNA expression shows a consistent pattern with micro-CT angiographic parameters with an increase at week 2 (day 7–14) and a decrease in week 3 (day 15–28).
