# Supplementary material for: Temporal Pattern of micro-CT Angiography Vascular Parameters and VEGF mRNA Expression in Fracture Healing: a Radiograph and Molecular Comparison
Source: Eur J Dent. 2023 Jan 30;17(2):283–95. doi: 10.1055/s-0042-1757466 (PMC10329534; doi:10.1055/s-0042-1757466)
Supplement: Supplementary file 1 — Supplementary Material [file 10-1055-s-0042-1757466-s2241994.pdf]

Supplementary Table S1 Data characteristics of animal models used in included studies

Included studies	Species	Sex	Age (wk)	Weight (g)	Number of samples (per timepoint)
Chen et al[25] (2017)	C57BL/6	Mix	8	–	12
Cheung et al[26] (2012)	Sprague–Dawley	Female	24	200–225	6
Cottrell et al[27] (2014)	Sprague–Dawley	Female	12	250–300	6
Ding et al[28] (2010)	C57BL/6J	Female	6	–	6
Ding et al[29] (2011)	C57BL/6J	Male	6	25	10
Gilbert et al[30] (2015)	Brown Norway	Male	–	–	2-4
He et al[31] (2012)	C57BL/6J	Female	18	–	4
He et al[32] (2011)	C57BL/6	Female	18	–	4
Hurley et al[33] (2016)	Vector mice	Female	8–10	–	–
Kidd et al[34] (2010)	Wistar	Female	20	270–360	8
Li et al[35] (2019)	C57BL/6	–	8	–	3
Li et al[36] (2012)	Rat	–	–	–	7
Li et al[37] (2018)	Sprague–Dawley	Male	4	100–130	–
Liu et al[38] (2017)	Sprague–Dawley	–	12-20	350–500	4–5
Macdonald and Shefelbine[15] (2013)	C57BL/6	Male	18-22	–	4–5
Martinez et al[39] (2010)	Wistar	Male	12	–	8
Matsumoto et al[40] (2013)	Sprague–Dawley	Female	28-32	–	16–18
McCabe et al[41] (2013)	Sprague–Dawley	Female	20	250–290	6
Minkwitz et al[42] (2015)	Sprague–Dawley	Female	20	250–290	5–6
Qiao et al[43] (2019)	Sprague–Dawley	Male	72–80	485 ± 60	8
Reumann et al[44] (2010)	C57BL/6N	Male	10–12	24 ± 2	5
Suen et al[45] (2014)	Sprague–Dawley	Male	24	–	6
Sun et al[46] (2012)	Sprague–Dawley	Female	24	220	4
Wang et al[47] (2012)	C57BL/6J	–	10–12	20–30	5–7
Wilson et al[48] (2015)	C57BL/6	Male	13–14	–	3
Yin et al[49] (2014)	C57/BL6	–	10–12	–	–
Yuasa et al[50] (2014)	C57/B6	Male	8	–	6–11
Zhao et al[23] (2014)	Sprague–Dawley	Male	6	200	6

Supplementary Table S2 Data characteristics of fracture conditioning in included studies

Included studies	Fracture type	Conditioning method	Area	Fixation
Chen et al[25] (2017)	Open	Bur ⌀ 0.5 mm	Tibial plate	25G needle
Cheung et al[26] (2012)	Closed Transversal	–	Mid-shaft of right femur	–
Cottrell et al[27] (2014)	Closed	Three-point bending device	Diaphysis of right femur	Stainless steel pin ⌀ 0.71 mm
Ding et al[28] (2010)	Closed Transversal	Fracture apparatus	Mid-shaft of right femur	25G needle
Ding et al[29] (2011)	Open Transversal	Sharp scissors	Right femur	Intramedullary pin ⌀ 0.8 mm

 © 2023. The Author(s).

Supplementary Table S2 (*Continued*)

Included studies	Fracture type	Conditioning method	Area	Fixation
Gilbert et al[30] (2015)	Comminuted	Dropping down a 500-g weight from the height of 25 cm (fracture apparatus)	Mid-shaft of right femur	Kirschner wire ⌀ 1.6 mm
He et al[31] (2012)	Open Transversal	Osteotomy with bone saw	Mid-shaft femur	Kirschner wire ⌀ 1.2 mm
He et al[32] (2011)	Open	Bur ⌀ 0.8 mm	Diaphysis of right femur	–
Hurley et al[33] (2016)	Closed Transversal	Bonnarens-Einhorn modification using drop-weight blunt guillotine apparatus	Mid-diaphysis of right tibia	26G needle
Kidd et al[34] (2010)	Fatigue fracture	Giving repetitive weight (stress fracture) using 17 N force machine	Right ulna	–
Li et al[35] (2019)	–	Three-point bending apparatus	Femur	23G needle
Li et al[36] (2012)	Open	Segmental defect ⌀ 5 mm	1/3 medial of femur	Miniplate
Li et al[37] (2018)	Open	Carborundum disc	Right tibia	K-wire
Liu et al[38] (2017)	Open	Three-point bending apparatus	Mid-diaphysis of right femur	23G pin
Macdonald and Shefelbine[15] (2013)	Open	Osteotomy using fine hand saw	Mid-shaft of left femur	External stainless steel pins fixation
Martinez et al[39] (2010)	Incomplete non-displaced	Cyclically loaded at peak forces ranging from 3.75 to 4.10 N	Right ulna	–
Matsumoto et al[40] (2013)	Open	Bur ⌀ 0,9 mm, 11.000 RPM	Right ibia	–
McCabe et al[41] (2013)	Closed	Bonnarens-Einhorn modification (3-point bending)	Mid-shaft of bilateral femur	Intermedullary pin 1.3 mm
Minkwitz et al[42] (2015)	Closed	Fracture apparatus	Right tibia	Kirschner wire ⌀ 1 mm
Qiao et al[43] (2019)	Closed	Drop-weight blunt guillotine apparatus	Left tibia	Kirschner wire ⌀ 0.8 mm
Reumann et al[44] (2010)	Open	Osteotomy, cutting vertically the ribs with scissors	8th ribs	–
Suen et al[45] (2014)	Open	Osteotomy using circular saw ⌀ 1.6 cm	Mid-shaft of right femur	Kirschner wire ⌀ 1.2 mm
Sun et al[46] (2012)	Closed	Three-point bending apparatus	Right femur	Kirschner wire ⌀ 1.2 mm
Wang et al[47] (2012)	Open	Trephine ⌀ 1.8 mm	Calvaria	–
Wilson et al[48] (2015)	Closed Transversal	Bonnarens–Einhorn modification (fracture apparatus)	Femur	Stainless steel pin
Yin et al[49] (2014)	Open	Einhorn device	Mid-diaphysis of femur	25G needle
Yuasa et al[50] (2014)	Open	Osteotomy	Mid-shaft of femur	23G pin
Zhao et al[23] (2014)	Transversal	–	Mid-diaphysis of right tibia	Not used

 © 2023. The Author(s).

Supplementary Table S3 Data characteristics of micro-CT angiography analysis

Included studies	Contrast agent	Micro-CT setting	Scanning area	VOI	Micro-CT Brand	Bone decalcification	Segmentation	Analysis software	Reconstruction software
Chen et al[25] (2017)	Silicon rubber compound containing lead chromate	10.5 µm	–	–	Viva MicroCT (Scanco Medical AG, Switzerland)	√	Threshold ≥ 100	–	–
Cheung et al[26] (2012)	Microfil MV-117	–	3.7 mm proximal and distal from the fracture line	–	VivaCT 40 (Scanco Medical, Switzerland)	√	Threshold binarisasi	–	–
Ding et al[28] (2010)	Microfil MV-122	10,5 µm isotropic voxel	–	Surround the callus border of the 2D slice image	GE eXplore Locus Sp Micro-CT	√	Threshold	Built-in software	Built-in software
Ding et al[29] (2011)	Microfil	10.5 µm	1 cm from fracture area	–	Scanco MicroCT 80 (Scanco Medical, Switzerland)	√	–	–	–
Gilbert et al[30] (2015)	Microfil MV-122	16 µm	–	500 slices at the center of the defect	VivaCT 40 (Scanco Medical, Switzerland)	√	Threshold	–	–
He et al[31] (2012)	Microfil 117	10.5 µm, 70 kV, 114 uA	Entire femur	400 slices at the fracture center	VivaCT 40 (Scanco Medical, Switzerland)	√	Global threshold 2D images	Built-in software	Built-in software
He et al[32] (2011)	Microfil 117	10.5 µm, 70 kV, 114 uA	–	Defect area	VivaCT 40 (Scanco Medical)	√	Global threshold 2D images	Built-in software	Built-in software
Li et al[35] (2019)	Microfil	18 µm, 80 kV, 112 uA	–	Based on the callus from the post-decalcification image	SkyScan 1172 (Bruker-microCT, Kontich, Belgium)	√	–	–	Nrecon software (v.1.6.9.4; Bruker)
Liu et al[38] (2017)	Microfil MV-120	6 µm, 45 kV, 177 uA, 300 ms	6.3 mm (1,050 slices) from the center of the fracture line	Based on the callus from the post-decalcification image	VivaCT 40 (Scanco Medical, Switzerland)	√	Threshold = lower 200-upper 1400, gaus sigma = 1,2, gaus support = 2	–	Scanco software (uCT Ray v4.0)
Macdonald and Shefelbine[15] (2013)	Microfil MV-120	21 µm, 180 kV, 133 mA	Entire femur approximately 30 mm (1,450 slices)	Approximately 8 mm around the fracture area (between the proximal & distal pins)	HMX ST22 (X-Tel System Ltd. Tring, UK)	X	Automatic binarization threshold	Fiji with a particle counter feature	Fiji Image Manipulation Software with trainable segmentation features
Matsumoto et al[40] (2013)	ZrCA (zirconium dioxide)	2.74 µm per voxel, 16-bit resolution	–	–	Synchotron radiation micro-CT beamline 20B2 (Harima, Japan)	X	Grayscale dilation, subtraction and 3 × 3 × 3 voxel filtration of a 17.9 keV image	BoneJ plugin 1.3.5 for ImageJ	–
Minkwitz et al[42] (2015)	Microfil MV-122	8.72 µm, 60 kV, 164 µA	–	Based on a 2D post decalcification slice imaging	SkyScan 1172 (Bruker-microCT, Kontich, Belgium)	√	Global threshold	Amira 5.7.0, Visage Imaging	Amira 5.7.0, Visage Imaging
Suen et al[45] (2014)	MicrofilMV-117	–	6 mm (300 slides) from the osteotomy line	–	VivaCT 40 (Scanco Medical, Switzerland)	√	Threshold 100 HU	ImageJ	–

 © 2023. The Author(s).

Supplementary Table S3 (*Continued*)

Included studies	Contrast agent	Micro-CT setting	Scanning area	VOI	Micro-CT Brand	Bone decalcification	Segmentation	Analysis software	Reconstruction software
Sun et al[46] (2012)	Microfil MV-117	16 µm per voxel	3.7 mm proximal and distal to the fracture line	–	VivaCT 40 (Scanco Medical, Switzerland)	√	–	–	–
Yin et al[49] (2014)	Microfil MV-122	10.5 µm	Entire femur	Based on the post decalcification 2D slice image/a little area around the callus	VivaCT 40 (Scanco Medical, Swizerland)	√	Global threshold based on intensity value	–	–
Yuasa et al[50] (2014)	Microfil MV-122	Resolution 20 µm isotropic voxel	Entire femur	–	µCT40 (Scanco Medical-AG, Switzerland)	√	Threshold with software	–	Scanco-Medical Evaluation Script
Zhao et al[23] (2014)	Barium sulfate	Resolution 9 µm, 70 kV, 142 uA	Entire posterior limb	4 mm from the middle of the tibial diaphysis fracture	SkyScan 1076 (Belgium)	√	–	CTAn (v.2.6)	Nrecon software (v.1.6.4.6)

Note Sign (-) does not have a clear description, sign (√) is carried out, sign (X) is not carried out

 © 2023. The Author(s).

Supplementary Table S4 Data characteristics of VEGF mRNA expression analysis

Included studies	Total RNA extraction method	Quantification/normalization
Cottrell et al[27] (2014)	Callus tissue was processed using liquid nitrogen, TRIzol, and the Qiagen RNeasy kit	Measure the relative expression Ct (1/ Cт) with internal GAPDH controls
Hurley et al[33] (2016)	The callus tissue was processed using TRIzol	Measure the relative expression of the Pfaffl method with internal control β-action mRNA
Kidd et al[34] (2010)	Ulna was ground into a fine powder using liquid nitrogen then homogenized using TRIzol	Measure the relative expression of the 2^{-Ct} method with internal GAPDH control was then compared with the contralateral site
Li et al[36] (2012)	The fracture tissue was processed using liquid nitrogen and TRIzol	Measure the cumulative expression with the internal control 18S mRNA (r18s)
Li et al[37] (2018)	Callus tissue using TRIzol and purified by UV spectrophotometer	Measure the relative expression of the 2^{-Ct} method with internal control β-actin and non-fractured tissue (before surgery)
Martinez et al[39] (2010)	Ulnae tissue was pulverized with liquid nitrogen and processed using TRIzol, RNA was purified with an Rneasy Mini Kit	Measure relative expression using the internal control Hprt (Ct) and non-fractured contralateral tissue (Ct)
McCabe et al[41] (2013)	The callus tissue at weeks 1 and 2 fractures (1.5 cm ROI) was isolated by deep pulverization using TRIzol	Measure the relative expression of the Ct method with normalization using the internal control 18S mRNA (r18s)
Minkwitz et al[42] (2015)	Fractured tissue (5 mm proximal and distal), pulverized with liquid nitrogen and extracted using the TriFast-method with a combination of Precellys homogenizer	Measure the relative expression of the Ct method with internal control cyclophilin A (Ppia) and non-fracture groups
Qiao et al[43] (2019)	Callus tissue in fractures using TRIzol according to the manufacturer's instructions	Measure the relative expression using the internal GAPDH control
Reumann et al[44] (2010)	The 8th rib and involved a little part of the 7th and 9th ribs, pulverized using TRIzol	Measure the relative expression with internal normalization of the GAPDH control and then compare with the non-fracture group
Wang et al[47] (2012)	The callus tissue was mashed using liquid nitrogen and extracted by the RNAqueous-Micro kit	Measure the relative expression with the internal normalization of GAPDH control and normal groups
Wilson et al[48] (2015)	The callus tissue in the fracture was processed using liquid nitrogen and TRIzol	Measure the relative expression with normalized internal control of β-actin (Ct) and correlation with the contralateral network (Ct)

 © 2023. The Author(s).
